# Prognostic Value of Pretreatment Serum Transthyretin Level in Patients with Gastrointestinal Cancers

**DOI:** 10.1155/2019/7142065

**Published:** 2019-06-03

**Authors:** Hongliang Luo, Jun Huang, Zhengming Zhu, Peiqian Zhu

**Affiliations:** Department of General Surgery, The Second Affiliated Hospital of Nanchang University, Nanchang, 330000 Jiangxi Province, China

## Abstract

**Background:**

Many studies have shown the link between the pretreatment serum transthyretin and prognosis in gastrointestinal (GI) cancers. However, based on the conclusion, the initial findings were inconsistent. Hence, this meta-analysis was performed to identify the prognostic values of the pretreatment serum transthyretin in GI cancers.

**Methods:**

Previous studies published before November 2018 were collected from a comprehensive literature search of several databases. The pooled hazard ratios (HRs) and 95% confidence intervals (CIs) were applied in the assessment of the intensity of associations. Also, the publication bias and the robustness of merged data were assessed. All statistical analyses were undertaken using STATA/SE 14.1.

**Results:**

The combined data indicated that the pretreatment serum transthyretin level was related to the prognosis in GI cancers. The group with reduced pretreatment transthyretin had a significantly worse overall survival (OS) (HR = 1.71, 95% CI: 1.37-2.05). The subgroup analysis for OS further showed the predictive value of transthyretin. In addition, the low serum transthyretin level could be an unfavorable factor for recurrence-free survival (RFS) or progression-free survival (PFS) (HR = 1.66, 95% CI: 1.14-2.18) in GI cancers.

**Conclusion:**

The low pretreatment serum transthyretin level implies an unfavorable prognosis for patients with GI cancers. The monitoring of pretreatment transthyretin level could contribute to the risk stratification and individualized therapy in GI cancers.

## 1. Introduction

Transthyretin, also known as prealbumin, is mainly synthesized by the hepatocyte and less likely caused by hepatic disease compared to other serum proteins [1, 2]. Transthyretin has a relatively short half-life with a high percentage of essential amino acids [3]. Serum transthyretin has been found related to several cases of malnutrition. It is the earliest laboratory indicator that is used to evaluate nutritional status [4, 5]. In addition, transthyretin is correlated with clinical outcomes in various diseases [6–8]. Recently, several studies reported that transthyretin might be a useful prognostic tumor marker [9, 10]. A high level of serum albumin is found with shorter survival rates in glioblastoma patients [9]. The pretreatment transthyretin level is seen to be an independent prognostic indicator among patients with metastatic renal cell carcinoma [10]. However, the prognostic significance of pretreatment transthyretin in patients with digestive cancer is inconsistent [11–14]. Some studies suggested that pretreatment transthyretin is significantly linked to the prognosis of patients with gastrointestinal (GI) cancers, but some have failed to get similar results. For example, serum transthyretin is found to be a significant independent factor for overall survival (OS) in the intrahepatic cholangiocarcinoma and hepatocellular carcinoma [11, 12]. However, no significant connection is established between the transthyretin level and OS among patients with gastric cancer [14]. Thus, to clarify the predictive value of this potential digestive cancer biomarker, a systematic review and meta-analysis are conducted to evaluate the relationships between transthyretin and prognosis in the digestive cancers.

## 2. Material and Methods

### 2.1. Search Strategy

A systematic literature search of PubMed, Embase, and Web of Science (up to November 1, 2018) was carried out by combining all related terms such as “transthyretin or transthyretin” and “tumor or cancer or carcinoma” and “survival or prognosis or outcome.” The articles published in the English language were selected, and we also manually searched for the relevant studies in the references of eligible publications.

### 2.2. Inclusion and Exclusion Criteria

The inclusion criteria included the following: (1) All patients were pathologically shown as primary GI cancers. (2) The correlation between pretreatment serum transthyretin and OS or recurrence-free survival (RFS) or progression-free survival (PFS) was analyzed. (3) Other related cases were classified into low and high transthyretin level groups. (4) The cut-off value for the high transthyretin level was provided.

The exclusion criteria were the following: (1) studies that focused on the relationship between transthyretin and cancers from the nondigestive system; (2) no available usable data of the hazard ratios (HRs) and 95% confidence intervals (CIs); (3) authors' usage of abstracts, reviews, letters, editorials, and case reports; (4) related overlapping or duplicate studies.

### 2.3. Data Extraction

Data separately extracted by two authors from each study included the following: the full name of the first author, the publication year, country, and type of study, along with time, total number of recruited cases, age distribution, number of males, clinical stages, cut-off selection, cut-off value, treatment methods, follow-up time, and the outcomes of OS and RFS/PFS. HRs and 95% CIs were directly selected from the multivariate or univariate analysis. The HR > 1 indicated worse OS or RFS/PFS for the patients with low serum transthyretin. A quality assessment method was used from a study by Lin et al. [15] in the meta-analysis. This scale had a total of nine items on the methodology with a final score ranging from 0 (lowest) to 9 (highest).

### 2.4. Statistical Analysis

All data analyses were held using the STATA/SE 14.1. Synthesized HRs and 95% CIs were used to assess the relation of pretreatment serum transthyretin with OS and RFS/PFS. In addition, the prognostic values of serum transthyretin in GI cancers were further assessed by conducting subgroup evaluation. Chi-square-based *Q* and *I* ^2^ tests were used to evaluate the heterogeneity among related studies. The heterogeneity was considered significant if *I* ^2^ > 50% or *P* < 0.01, then the random effects model was employed. Otherwise, the fixed effects model was selected. To test the stability of the combined results, sensitivity analysis was conducted by evaluating each study carefully. Potential publication bias was assessed using Begg's plots and tests.

## 3. Results

### 3.1. Study Selection and Characteristics

**Figure 1:**
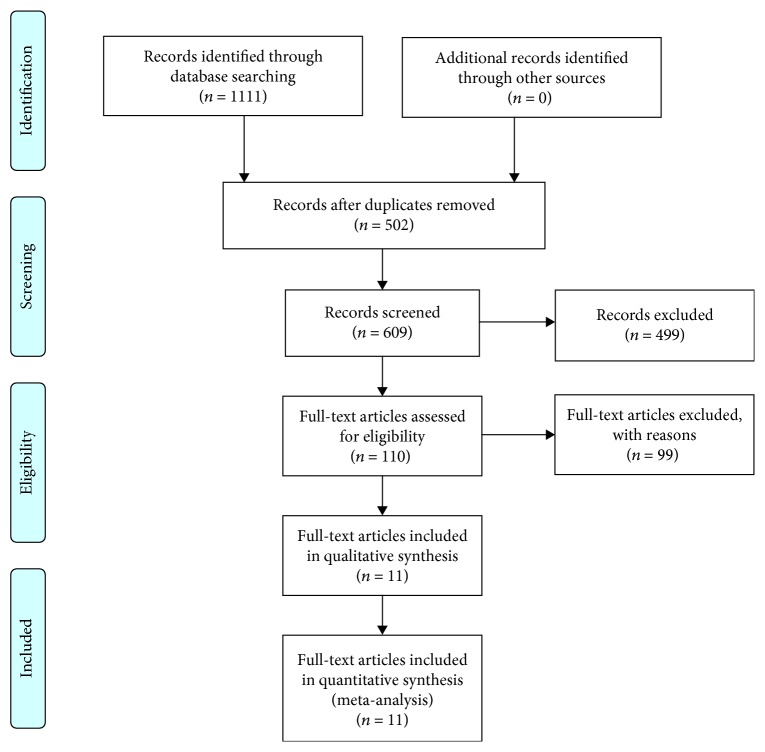
Flow diagram of literature selection.

**Figure 2:**
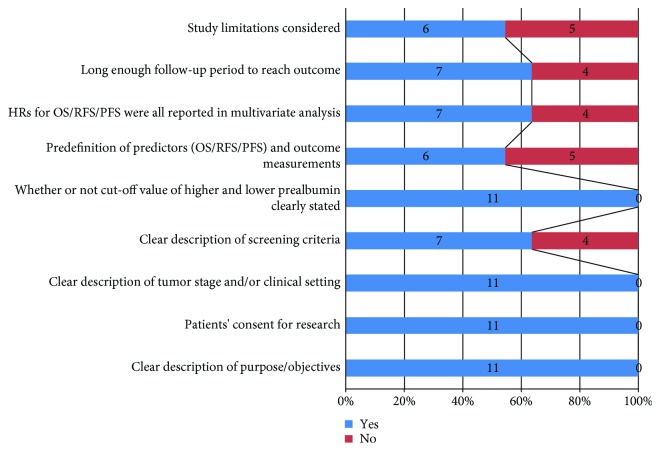
Quality assessment of eleven eligible cohort studies.

Literature retrieval strategies are seen in Figure 1. Finally, a total of 11 full-text articles in English were included [11–14, 16–22]. They all reported the connection between pretreatment transthyretin and survival outcomes in digestive cancers. The enrolled studies were carried out in China (*n* = 6), Japan (*n* = 4), and Romania (*n* = 1). The median population size was 110 (range: 25-1483). Five types of digestive cancers were analyzed, including adenocarcinoma of the esophagogastric junction (AEG), gastric cancer (GC), colorectal cancer (CRC), intrahepatic cholangiocarcinoma (ICC), and hepatocellular carcinoma (HCC). All cohort studies identified the relevance between pretreatment serum transthyretin and OS. Three studies focused on RFS. One study explored the relationship between the transthyretin level and PFS. The cut-off values for a high level of transthyretin varied from 114 mg/L to 400 mg/L while the cut-off values were mainly determined by the receiver operating characteristic (ROC) curves. The results of the quality assessment are shown in Figure 2 with a median score of 7 (ranges from 5 to 9, Table S1). The summary characteristics of eligible studies are detailed in Table 1.

## 4. Results of the Meta-Analysis

### 4.1. Transthyretin and OS

**Figure 3:**
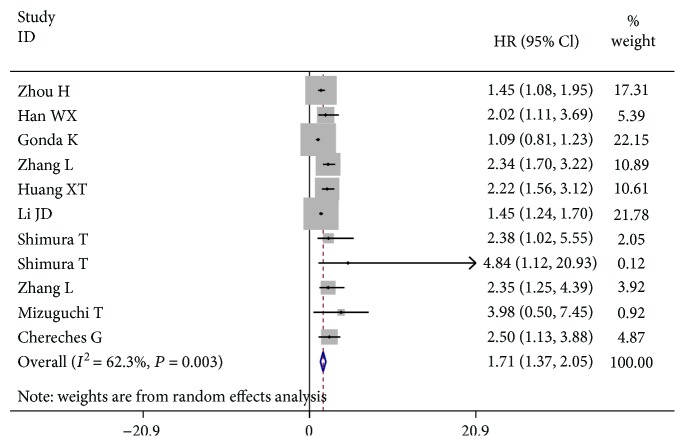
The prognostic value of the low pretreatment serum transthyretin level on OS in digestive cancers.

A total of 11 eligible studies were evaluated to identify the relationship between pretreatment serum transthyretin and OS of digestive cancers using HR data. In Figure 3, compared with patients in the high transthyretin group, the cases with a low transthyretin level had a worse OS, with a combined HR of 1.71 (95% CI: 1.37–2.05) in the random effects model (*P* = 0.003; *I* ^2^ = 62.3%). Also, an essential prognostic significance of transthyretin was highlighted in ICC (HR = 1.76, 95% CI: 1.02–2.51) in the random effects model (*P* = 0.090; *I* ^2^ = 65.1%), AEG (HR = 2.26, 95% CI: 1.60–2.91) in the fixed effects model (*P* = 0.683; *I* ^2^ = 0.0%), and HCC (HR = 1.48, 95% CI: 1.25–1.71) in the fixed effects model (*P* = 0.301; *I* ^2^ = 18.0%), but not in GC (HR = 1.10, 95% CI: 0.89–1.31) in the fixed effects model (*P* = 0.268; *s*).

### 4.2. Subgroup Analysis of OS

The significance of pretreatment serum transthyretin was further evaluated using subgroup analysis based on the disease type, treatment, cut-off value, clinical stage, follow-up, and analysis type (Table 2). A vital correlation was shown between the low transthyretin level and shorter OS for GI tract cancers (HR = 1.92, 95% CI: 1.11-2.73) and non-GI tract cancers (HR = 1.62, 95% CI: 1.29-1.96). Obviously, a decreased transthyretin was related to worse OS both in nonmetastatic patients and in mixed cases, with a combined HR estimate of 1.45 (95% CI: 1.22-1.68) and 1.97 (95% CI: 1.54-2.39), respectively. Furthermore, the low transthyretin level was notably associated with inferior OS in the subgroup with long-term follow-ups (≥5 years) (HR = 1.98, 95% CI: 1.46-2.49) and the patients treated with surgery (HR = 1.78, 95% CI: 1.45-2.12). Other subgroups stratified by cut-off value and analysis type also showed significant correlations between the pretreatment transthyretin level and OS in digestive cancers.

### 4.3. Transthyretin and RFS/PFS

**Figure 4:**
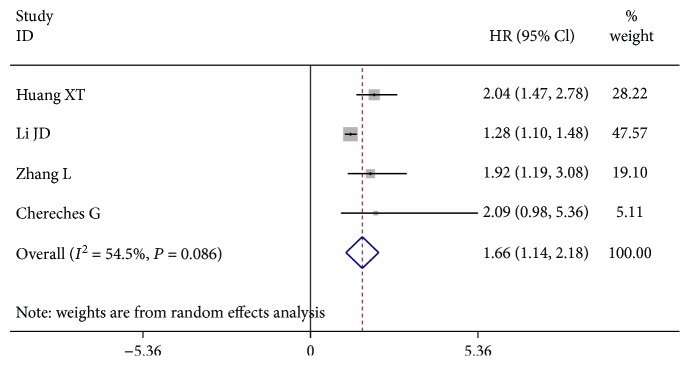
Pooled HR for the association between the transthyretin level and RFS/PFS.

Four cohort studies identified the HRs for the connection between the transthyretin level and RFS/PFS in the digestive cancers. In Figure 4, the patients with the low serum transthyretin level had a worse RFS/PFS compared to patients in the high serum transthyretin group (HR = 1.66, 95% CI: 1.14-2.18). The pretreatment decreased serum transthyretin, indicating a poor factor of RFS/PFS among patients with digestive cancers.

### 4.4. Publication Bias

**Figure 5:**
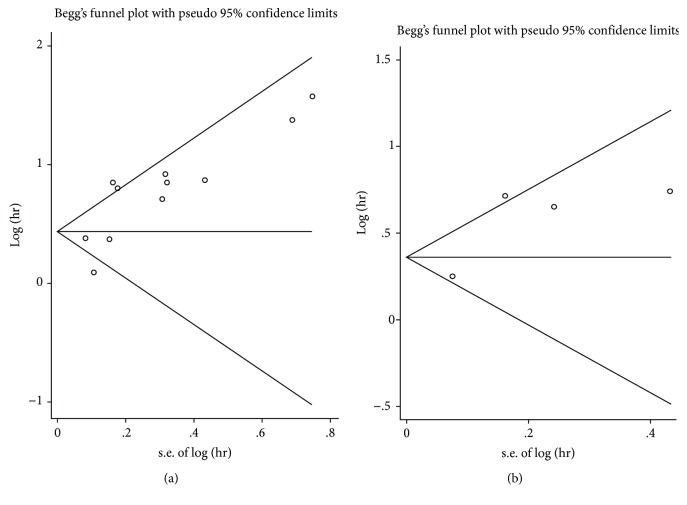
Publication bias assessment regarding OS (a) and RFS/PFS (b).

Begg's funnel plots are shown in Figure 5 while the *P* values in Begg's tests were higher than 0.05, indicating no significant publication bias was found among the studies.

### 4.5. Sensitivity Analysis

**Figure 6:**
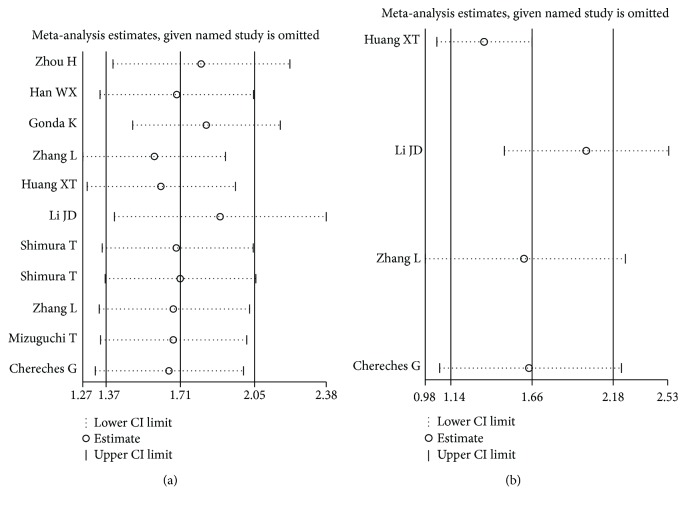
Sensitivity analysis of the OS (a) and RFS/PFS (b).

Sensitivity analysis was seen to have no clear variation in the overall HRs. The results were reliable and robust (Figure 6).

## 5. Discussion

Recently, many gastrointestinal tumor biomarkers are reported. However, some of them are confined to the tumor tissues [23–26]. Because the acquisition of the tumor tissue is invasive and quite hard to identify, this limits the application of these markers in the clinical practice. GI cancers, one of the most common and malignant tumors, often lead to poor prognosis [27–29]. The noninvasive, easily accessible factors are more conducive to risk stratification and prognosis assessment, which are useful for implementing an individualized treatment. Transthyretin is a protein that can be easily identified in the blood, inexpensive, and noninvasive. Also, it has attracted much attention for its stability and sensitivity [3, 30, 31]. At the same time, transthyretin is considered a good marker in assessing the patients' nutritional status. It has a much shorter half-life (2–3 days) and can be unaffected by hydration status [32–34]. However, some studies are against the use of transthyretin levels as nutrition markers and patients' outcomes [35–37]. For some treatments and pathological states, such as corticosteroid therapy, renal dysfunction, infection, physiological stress, and liver dysfunction, they can increase or decrease transthyretin levels [32]. Recently, more clinical studies also showed that serum transthyretin is related to patient prognosis in GI cancer and might serve as a promising novel prognosticator [20–22].

To the best of our knowledge, this meta-analysis for the first time systematically clarified the prognostic value of pretreatment serum transthyretin in patients with digestive cancer. In this paper, a pooled HR of 1.71 was taken with the corresponding 95% CI (1.37–2.05) for OS when all the currently available data were combined. The results indicated that low transthyretin was associated with a poor OS for digestive cancer. The predictive role of serum transthyretin was also seen in the specific types of digestive cancer, including ICC, AEG, and HCC. Furthermore, the subgroup analyses for OS were carried out. It was found that decreased transthyretin was an unfavorable indicator for both GI tract cancer and non-GI tract cancer. Also, it showed that a low transthyretin level was connected with shorter OS among the patients after surgery or several cases in the nonmetastatic stage or all stages. Meanwhile, the low transthyretin level might have bad effects on the long-term survival of digestive cancer patients. Similar results were also seen in the other two subgroup analyses through the cut-off value and analysis type. Additionally, decreased transthyretin was significantly correlated with inferior RFS/PFS in the digestive cancers (HR = 1.66, 95% CI: 1.14-2.18). Therefore, the pretreatment serum transthyretin might act as a useful prognostic marker that can be used to estimate the survival outcomes of the digestive cancers.

However, the results based on the analysis should be interpreted cautiously since there were several existing limitations. First, there was significant heterogeneity among the related studies. Although the random effects model was used with the pooled data, heterogeneity could be explained by the differences in the clinical pathological factors, such as age, tumor type, and disease stage. Second, several HRs and 95% CI were available in the univariate analysis but not in the multivariate analysis. Third, this meta-analysis only included published English studies. Fourth, the number of selected studies and enrolled cases was relatively limited while more studies with different populations are needed in the future. Finally, there was no consensus regarding the definition of the cut-off value for decreased serum transthyretin in the selected studies. A definitive cut-off value is highly recommended.

In summary, the study provided strong evidence that decreased pretreatment transthyretin was significantly related to poor clinical outcomes among patients with digestive cancers. Transthyretin could be used in clinical practice as a widely accepted, stable, and inexpensive nutritional indicator to evaluate the prognosis of digestive cancers. However, considering the limitations cited above, more well-designed and multicenter clinical studies should be conducted to further validate the predictive value of transthyretin in digestive cancer.

## Figures and Tables

**Table 1:** Main characteristics of all included cohort studies.

Study	Year	Cancer type	Country	Study type	Included time	Total sample	Age	No. of males (%)	Stage	Cut-off selection	Cut-off value	Treatment methods	Follow-up	End points (analysis type)
Zhou H	2015	ICC	China	R	2005-2009	370	NA	236 (63.8%)	Mixed	NA	170 mg/L	With surgery	<5 years	OS (M)
Mizuguchi T	2009	HCC	Japan	R	2001-2005	100	NA	76	Mixed	ROC	150 mg/L	With surgery	≥5 years	OS (U)
Han WX	2016	AEG	China	R	January and July 2010	101	Mean 65	80 (79.2%)	Mixed	ROC	200 mg/L	With surgery	≥5 years	OS (M)
Gonda K	2017	GC	Japan	R	2013-2015	110	Median 66.2	56 (50.9%)	Metastatic	NA	180 mg/L	No surgery	<5 years	OS (M)
Chereches G	2017	CRC	Romania	P	2012-2015	72	Median 60	58.3%	Metastatic	NA	400 mg/L	No surgery	<5 years	OS (U), PFS(U)
Zhang L	2017	AEG	China	R	2010.10-2011	355	Median 64	281 (79.2%)	Mixed	ROC	208.33 mg/L	With surgery	≥5 years	OS (M)
Huang XT	2018	ICC	China	R	2006-2017	276	Median 58	147 (53.3%)	Mixed	ROC	184 mg/L	With surgery	≥5 years	OS (U), RFS (U)
Li JD	2018	HCC	China	R	2001-2014	1483	Mean 51	1317 (89%)	No metastatic	NA	170 mg/L	With surgery	≥5 years	OS(M), RFS(M)
Shimura T	2018	GC	Japan	R	2011-2013	30	NA	21 (70%)	Mixed	ROC	228 mg/L	With surgery	≥5 years	OS (M)
Shimura T	2018	HCC	Japan	R	2011-2013	25	Mean 69.6	22 (88%)	No metastatic	ROC	114 mg/L	With surgery	≥5 years	OS (M)
Zhang L	2018	HCC	China	R	2011-2013	230	Mean 51.60	193 (83.90%)	Mixed	ROC	152.5 mg/L	With surgery	<5 years	OS (M), RFS (U)

P: prospective; R: retrospectively; ICC: intrahepatic cholangiocarcinoma; AEG adenocarcinoma of esophagogastric junction; GC: gastric cancer; CRC: colorectal cancer; HCC: hepatocellular carcinoma; OS: overall survival; RFS: recurrence-free survival; PFS: progression-free survival; ROC: receiver operating characteristic; U: univariate analysis; M: multivariate analysis: NA: not available.

**Table 2:** Stratified analysis of pooled HRs for digestive cancer patients with lower transthyretin.

Subgroup factor	Divided standard	No. of studies	Pooled HR (95% CI)	*P* value	Heterogeneity	
					*I* ^2^ (%)	*P*_het_
Cancer type	GI tract cancer	5	1.92 (1.11-2.73)	<0.001	74.1	0.004
	Non-GI tract cancer	6	1.62 (1.29-1.96)	<0.001	28.2	0.223

Cut-off value	<180 mg/L	5	1.47 (1.27-1.68)	<0.001	0.0	0.451
	≥180 mg/L	6	1.98 (1.26-2.69)	<0.001	76.4	0.001

Treatment	No surgery	2	1.62 (0.29-2.96)	NS	74.6	0.047
	With surgery	9	1.78 (1.45-2.12)	<0.001	33.4	0.150

Analysis type	UVA	3	2.35 (1.68-3.02)	<0.001	0.0	0.607
	MVA	8	1.53 (1.21-1.85)	<0.001	58.8	0.018

Follow-up	<5 years	4	1.46 (0.97-1.95)	NS	61.3	0.052
	≥5 years	7	1.98 (1.46-2.49)	<0.001	43.9	0.098

Clinical stage	Nonmetastatic	2	1.45 (1.22-1.68)	<0.001	0.0	0.503
	Metastatic	2	1.62 (0.29-2.96)	NS	74.6	0.047
	Mixed	7	1.97 (1.54-2.39)	<0.001	23.1	0.253

GI: gastrointestinal; HR: hazard ratio; 95% CI: 95% confidence interval; UVA: univariate analysis; MVA: multivariate analysis; NS: not significant.

## Supplementary Materials

Supplementary Materials Table S1: quality assessment of 11 studies included in the meta-analysis.
