# Supplementary material for: Prognostic Value of Pretreatment Serum Transthyretin Level in Patients with Gastrointestinal Cancers
Source: Dis Markers. 2019 Jun 3;2019:7142065. doi: 10.1155/2019/7142065 (PMC6589268; doi:10.1155/2019/7142065)
Supplement: Supplementary Materials — Table S1: quality assessment of 11 studies included in the meta-analysis. [file 7142065.f1.docx]

**Table S1** Quality assessment of 11 studies included in the meta-analysis.

| **Study [Reference]*** | **Cancer type** | **Clear description of purpose/objectives** | **Patients' consent for research** | **Clear description of tumor stage and/or clinical setting** | **Clear description of screening criteria** | **Whether or not cut-off value of higher and lower prealbumin clearly stated** | **Predefinition of predictors (OS/RFS/PFS) and outcome measurements** | **HRs for OS/RFS/PFS were all reported in multivariate analysis** | **Long enough follow-up period to reach outcome** | **Study limitations considered** | **Quality score (0 − 9)** |
| --- | --- | --- | --- | --- | --- | --- | --- | --- | --- | --- | --- |
| Zhou H | ICC | yes | yes | yes | yes | yes | yes | yes | no | yes | 8 |
| Mizuguchi T | HCC | yes | yes | yes | no | yes | no | no | yes | no | 5 |
| Han WX | AEG | yes | yes | yes | yes | yes | no | yes | yes | yes | 8 |
| Gonda K | GC | yes | yes | yes | no | yes | yes | yes | no | no | 6 |
| Chereches G | CRC | yes | yes | yes | yes | yes | no | no | no | no | 5 |
| Zhang L | AEG | yes | yes | yes | yes | yes | yes | yes | yes | yes | 9 |
| Huang XT | ICC | yes | yes | yes | yes | yes | yes | no | yes | no | 7 |
| Li JD | HCC | yes | yes | yes | yes | yes | yes | yes | yes | yes | 9 |
| Shimura T | GC | yes | yes | yes | no | yes | no | yes | yes | yes | 7 |
| Shimura T | HCC | yes | yes | yes | no | yes | no | yes | yes | yes | 7 |
| Zhang L | HCC | yes | yes | yes | yes | yes | yes | no | no | no | 6 |
